# Lactoferricin enables adenovirus infection of human skeletal muscle cells

**DOI:** 10.1038/s44298-025-00144-7

**Published:** 2025-08-19

**Authors:** Katarina Danskog, Nitesh Mistry, Carin Årdahl, Madeleine Durbeej, Mattias N. E. Forsell, Annasara Lenman, Niklas Arnberg

**Affiliations:** 1 Department of Clinical Microbiology, Umeå University, Umeå, Sweden; 2 Department of Experimental Medical Science, Lund University, Lund, Sweden; 3 The Laboratory for Molecular Infection Medicine Sweden (MIMS), Science for Life Laboratory (SciLifeLab), Umeå University, Umeå, Sweden; 4 Umeå Centre for Microbial Research (UCMR), Umeå University, Umeå, Sweden

**Keywords:** Vaccines, Virology

## Abstract

Although adenoviruses (AdVs) possess advantageous features as vectors, several challenges remain. These include a high prevalence of neutralizing antibodies against certain AdV types and the inability to efficiently transduce CAR-deficient cells and tissues. We showed previously that lactoferricin (Lfcin) enhances CAR-independent HAdV-C5 infection of epithelial and T-cells. Here, we assessed the ability of Lfcin to enable HAdV-C5 infection and transduction of human skeletal muscle cells. Lfcin increases HAdV-C5 infection and transduction of muscle myoblasts and myotubes by 10- to 30-fold. Enhanced infection correlates with increased cell binding, which differs mechanistically from that of coagulation factor X-mediated binding, as it remains unaffected by the removal of heparan sulfate. Additionally, Lfcin reduces the neutralizing effects of serum against HAdV-C5, suggesting it may shield key epitopes. By enabling viral binding to muscle cells and mitigating serum neutralization, Lfcin offers a novel strategy to improve the efficiency and durability of HAdV-C5-based gene delivery systems.

## Introduction

Adenovirus (AdV) based viral vectors are widely utilized for gene therapy, oncolytic therapy, and immunization, largely thanks to their ability to transduce both dividing and non-dividing cells, high transduction efficiency, and capacity to accommodate large transgenes. Several human adenovirus (HAdV)-based vaccines have been approved for protection against infectious diseases, such as Ebola^1^, and COVID-19^2–4^, and are also being evaluated as vaccines against for example, malaria^5^. AdV vectors are used for gene therapy with dystrophin in mice^6,7^, and with vascular endothelial growth factor in human myocardium^8^.

Many vectors are based on human adenovirus C5 (HAdV-C5). One major challenge with HAdV-C5 based vaccine vectors is the high prevalence of preexisting, neutralizing antibodies in the human population (over 50% seroprevalence against HAdV-C5 worldwide^9,10^). This can significantly reduce efficacy and as few as 1.4 anti-hexon IgG antibodies per capsid are sufficient to neutralize HAdVs^11,12^. Various strategies have been explored to evade antibody-mediated neutralization, primarily through modifications of the hexon protein, the major antigenic component of the capsid^13–17^. Additionally, HAdV-C5 is sequestered in the liver following intravenous administration, mediated by interactions between the hexon and coagulation factors, mainly Factor X (FX)^18,19^. Interestingly, FX can also partially block hexon-targeting antibodies from neutralization of HAdV-C5^20^. Another challenge with HAdV-based vectors for immunisation is the low or absent expression of the coxsackievirus and adenovirus receptor (CAR) on muscle cells^21^, which HAdV-C5 and most other HAdVs use for attachment and entry^22,23^. To bypass the need for CAR and increase transduction of muscle cells, attempts have been made to insert e.g., lysine residues in capsid proteins such as the knob domain of the fibre protein, which increase HAdV vector transduction of mature muscle tissue by 4-fold^24^; integrin-binding motifs in the hexon, which increased transduction 6- to 10-fold in smooth muscle cells^25,26^; and specific muscle-targeting peptides in the hexon, which increased transduction of murine muscle cells 20-fold^27^. Additionally, preincubation with the positively charged amines spermine and spermidine enhances HAdV-C5 vector transduction in mesenchymal stem cells^28^. It is also known that age plays a role as myocytes are more resistant to HAdV infection upon ageing^29–31^. This decreased infectivity may be attributed to a downregulation of RGD-binding integrins^29,32^, which are important for HAdV cell entry.

Previous data from our lab demonstrate that HAdV-C5 interacts with endogenous lactoferrin and lactoferrin-derived lactoferricin (Lfcin) peptides via the hexon protein^33,34^. This interaction mediates non-canonical CAR-independent binding to and infection of both epithelial cells, which express CAR only on the basolateral side in differentiated tissues, and T-lymphocytes, which lack CAR expression entirely. Lfcin binds to hexon hypervariable region 1 (HVR1), which is unusually long and negatively charged in species C HAdVs compared to other HAdV species^34^. Additionally, lactoferrin also enhances HAdV-C5 infection of dendritic cells, which express little to no CAR^35^. In lung epithelial cells, the pro-viral effect of lactoferrin is specific for species C, redirecting the virus to one or more unknown receptor(s)^33^, while in dendritic cells, it is pro-viral for species B, C, and D by redirecting the virus to Toll-like receptor 4 (TLR4)^35^.

Lactoferrin is an iron-binding glycoprotein present in many body fluids, e.g., constitutively secreted by the tear and mammary glands, and by neutrophils at sites of inflammation^36–39^. It is associated with the modulation of inflammatory responses and exerts anticancer properties^37,38,40,41^. Lactoferrin has also shown antiviral properties against several RNA- and DNA-viruses such as severe acute respiratory syndrome coronavirus 2 (SARS-CoV-2)^42,43^, RSV^44^ and herpes simplex virus 1 (HSV1)^45^. In mucosa, human lactoferrin is cleaved at the N-terminus by proteases, releasing Lfcin, a cationic 49 amino acid long peptide with similar properties as lactoferrin^46,47^. Lfcin is positively charged at neutral pH, and the positively charged residues are implied in the interaction with HVR1 of the HAdV-C5 hexon^48^. In further support of lactoferrin/Lfcin playing a role during HAdV infection, lactoferrin-secreting neutrophils can facilitate adenovirus entry and infection^49^.

Here, we investigated if Lfcin can modulate HAdV-C5 infection of human skeletal muscle cells and possibly prevent antibody-mediated neutralization. A better understanding of these interactions will help targeting of adenovirus-based vectors to relevant tissues and cells, such as muscle cells, and thereby improve applications such as vaccination.

## Results

### Lfcin enables species C HAdV infection of non-differentiated human skeletal myoblasts

**Fig. 1:**
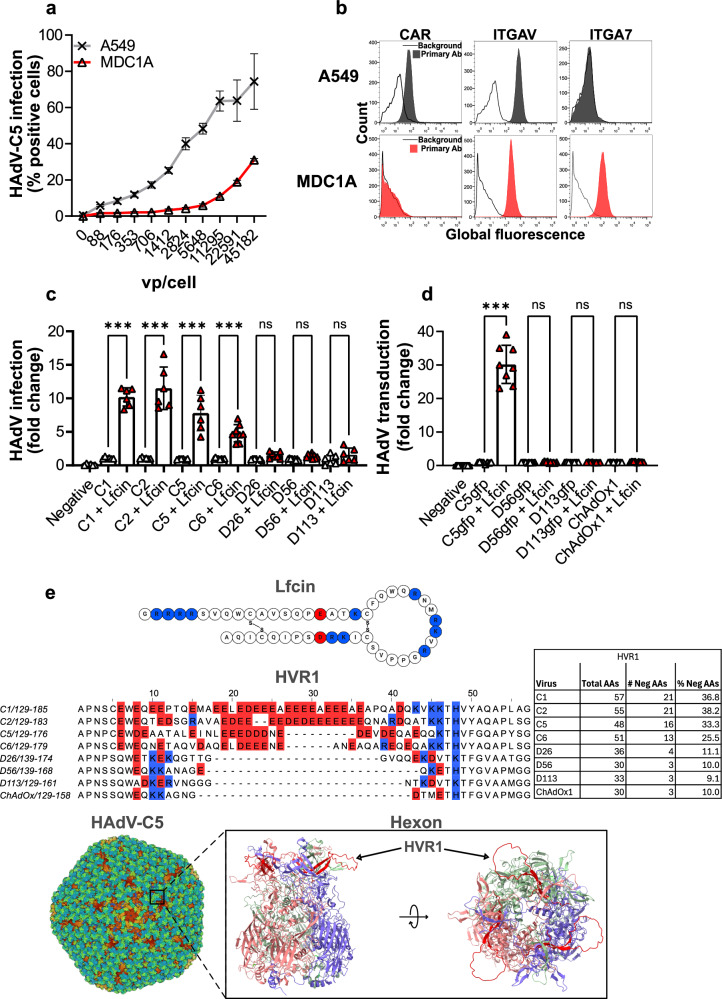
Lfcin enables HAdV-C5 infection of human skeletal muscle cells. **a** Analysis of titration of HAdV-C5 infection in A549 and myoblasts, stained for HAdV capsid protein and cell nuclei (vp: physical virus particles). **b** Flow cytometry analysis of cell surface expression of HAdV receptors on A549 and myoblasts (CAR: coxsackie and adenovirus receptor, ITGAV: Integrin αv, ITGA7: Integrin α7). **c** Analysis of HAdV-C (HAdV-C1 (6000 vp/cell), −C2 (20 000 vp/cell), −C5 (6000 vp/cell), − C6 (20 000 pb/cell) and HAdV-D (HAdV-D26) (12 000 vp/cell), −D113 (30 000 vp/cell) infection of myoblasts, stained for HAdV capsid protein. Fold change represents infection in the presence versus the absence of Lfcin. **d** Analysis of AdV vector (HAdV-C5-eGFP (600 vp/cell), HAdV-D56-eGFP (2000 vp/cell), HAdV-D113-eGFP (20,000 vp/cell), and ChAdOx1-Spike (20,000 vp/cell) transduction of myoblasts, analysed for GPF expression or stained for SARS-CoV-2 spike protein. Fold change represents transduction in the presence versus the absence of Lfcin. **e** Amino acid sequence and structure of Lfcin, and alignment of HVR1 amino acid sequences of HAdV-C1, −C2, −C5, −C6, −D26, −D56, −D113 and ChAdOx1. Residues marked in blue are positively charged and residues in red are negatively charged. The total number of residues, number of negatively charged residues and percent negative residues within HVR1 are listed in the table. Structures of the HAdV-C5 capsid (PDB: 6B1T) and the trimeric hexon (PDB: 3TG7). In red is a prediction of the HVR1 flexible loop modelled with AlphaFold3^74^ and rendered using ChimeraX^75^. Data from **a**, **c**, and **d** and are from at least two independent experiments, presented as mean ± SD. In **c** and **d**, statistical significance was determined with two-way ANOVA; ns, not significant; *** *P* < 0.001.

To investigate whether Lfcin influences adenoviral infection of skeletal muscle cells, we examined its effect on HAdV-C5 infection of myoblasts using a human immortalized myoblast cell line. We observed that a high viral load was required for infection (Fig. 1a). Flow cytometry analysis confirmed the lack of surface CAR and showed moderate expression levels of the HAdV-C5 entry receptor integrin alpha v (ITGAV), along with high levels of the muscle cell marker integrin alpha 7 (ITGA7) (Fig. 1b). As expected, A549 cells, which are commonly used for adenovirus studies and for virion propagation, expressed both CAR and ITGAV, but lacked expression of ITGA7. Multiple HAdV types of e.g., species C and D as well as non-human primate AdVs, have been used as vaccine vectors^50^. Here, we inoculated myoblasts with the four wild type species C HAdVs (HAdV-C1, −C2, −C5, −C6) as they all contain a negatively charged hypervariable region 1 (HVR1), assumed to interact with Lfcin; species D types of relevance for vaccination (HAdV-D26, −D56, and −D113); HAdV-based vectors expressing GFP (based on HAdV-C5, −D56, and −D113), as well as the chimpanzee adenovirus ChAd-Y25 vector expressing SARS-CoV-2 spike protein (ChAdOx1-Spike; as this AdV has also been used for vaccination), in absence and presence of Lfcin. We observed a 5- to 15-fold Lfcin-dependent increase in infection/transduction by species C HAdVs and corresponding vectors, but did not see any change in infection/transduction by other wild-type AdVs or vectors (Fig. 1c, d). Infection increased to 100% for HAdV-C wild-type viruses when Lfcin was present (from 10% baseline infection) (Supplementary Fig. 1), and transduction to 40% for the HAdV-C vector (from 1% baseline transduction) (Supplementary Fig. 2). Amino acid alignment analysis showed that hypervariable region 1 (HVR1) of HAdV-D26, −D56, −D113 and ChAdOx1 are remarkably shorter and contain clearly fewer negatively charged residues compared to species C HAdVs (Fig. 1e), suggesting that positively charged Lfcin enables AdV infection/transduction of myoblasts through charge-dependent interactions with negatively charged HVR1. In support of this assumption, HAdV-C1 and C2, which have the longest and most negatively charged HVR1, infected cells more efficiently in the presence of Lfcin as compared to HAdV-C5 and C6 (Fig. 1c), whose HVR1 regions are relatively short and less negatively charged (Fig. 1e). In addition, AlphaFold modelling and surface charge prediction of HVR1 further shows that HAdV-C1, C2, and C5 have a stronger negative charge than HAdV-C6, and that the HVR1 of HAdV-D26 is minimally exposed and carries an overall positive charge (Supplementary Fig. 3).

### Lfcin enables HAdV-C5 infection of differentiated human skeletal myotubes

**Fig. 2:**
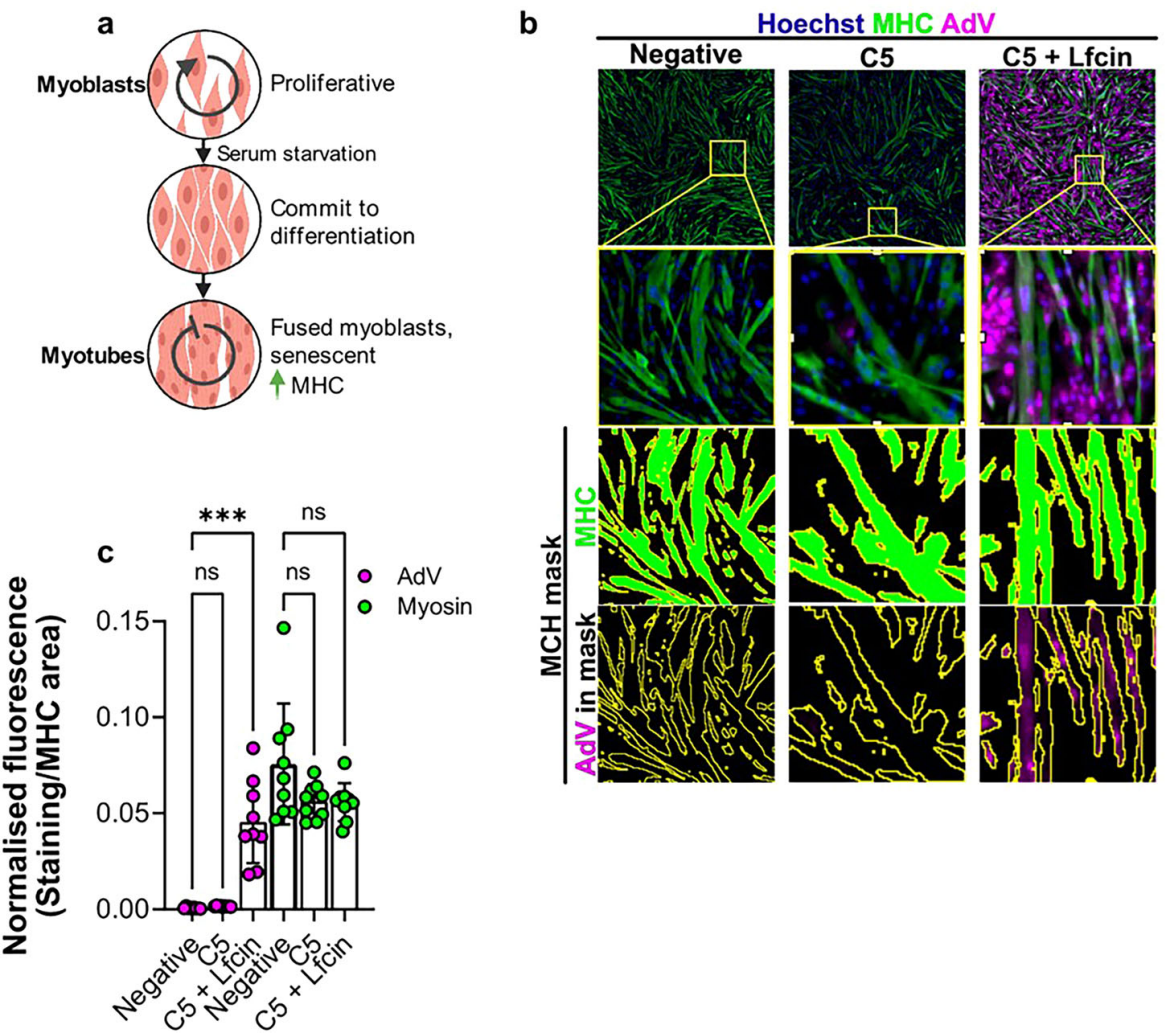
Lfcin enables HAdV-C5 infection in human skeletal myotubes. **a** Schematic image of the differentiation of myotubes from myoblasts. **b** Analysis of HAdV-C5 infection (15 000 vp/cell) of differentiated myotubes in the presence of 2 µM Lfcin, stained for HAdV capsid protein (magenta), myosin (green) and cell nuclei (blue). A mask was created based on MHC expression and fluorescence intensity from the HAdV-C5 staining and the myosin staining was calculated within the mask. **c** Quantification of fluorescence intensity of HAdV-C5 positive staining (pink) and myosin staining (green) within the MHC mask as shown in (**b**). Quantification was done for entire wells (0.32 cm^2^) in triplicates. Data is from three independent experiments and is shown as mean fluorescence intensity divided by area (mm^2^) ± SD. Statistical significance was determined ANOVA; ns, not significant; *** *P* < 0.001.

Muscle tissue primarily consists of long multinucleated muscle fibres with terminally differentiated myotubes. Myoblasts are proliferating single-nucleated cells, but when cultured in low-serum medium for five days, these cells differentiate into long multinucleated myotubes, which more closely resemble natural muscle fibres (Fig. 2a). We infected differentiated myotubes with HAdV-C5 in the presence or absence of Lfcin to observe whether Lfcin would increase infection in a similar way as in myoblasts. To distinguish myotubes from non-differentiated myoblasts, we stained cells with an anti-myosin heavy chain (MHC) antibody, as MHC is upregulated in myotubes (shown as green in Fig. 2b). We then applied an MHC mask and quantified the average fluorescence intensity of HAdV-C5 staining specifically within myotubes. In the absence of Lfcin, HAdV-C5 infection resulted in minimal (magenta) fluorescence; however, in the presence of Lfcin, fluorescence intensity increased by an average of 30-fold (Fig. 2b, c).

### Lfcin promotes viral entry into human skeletal myoblasts

**Fig. 3:**
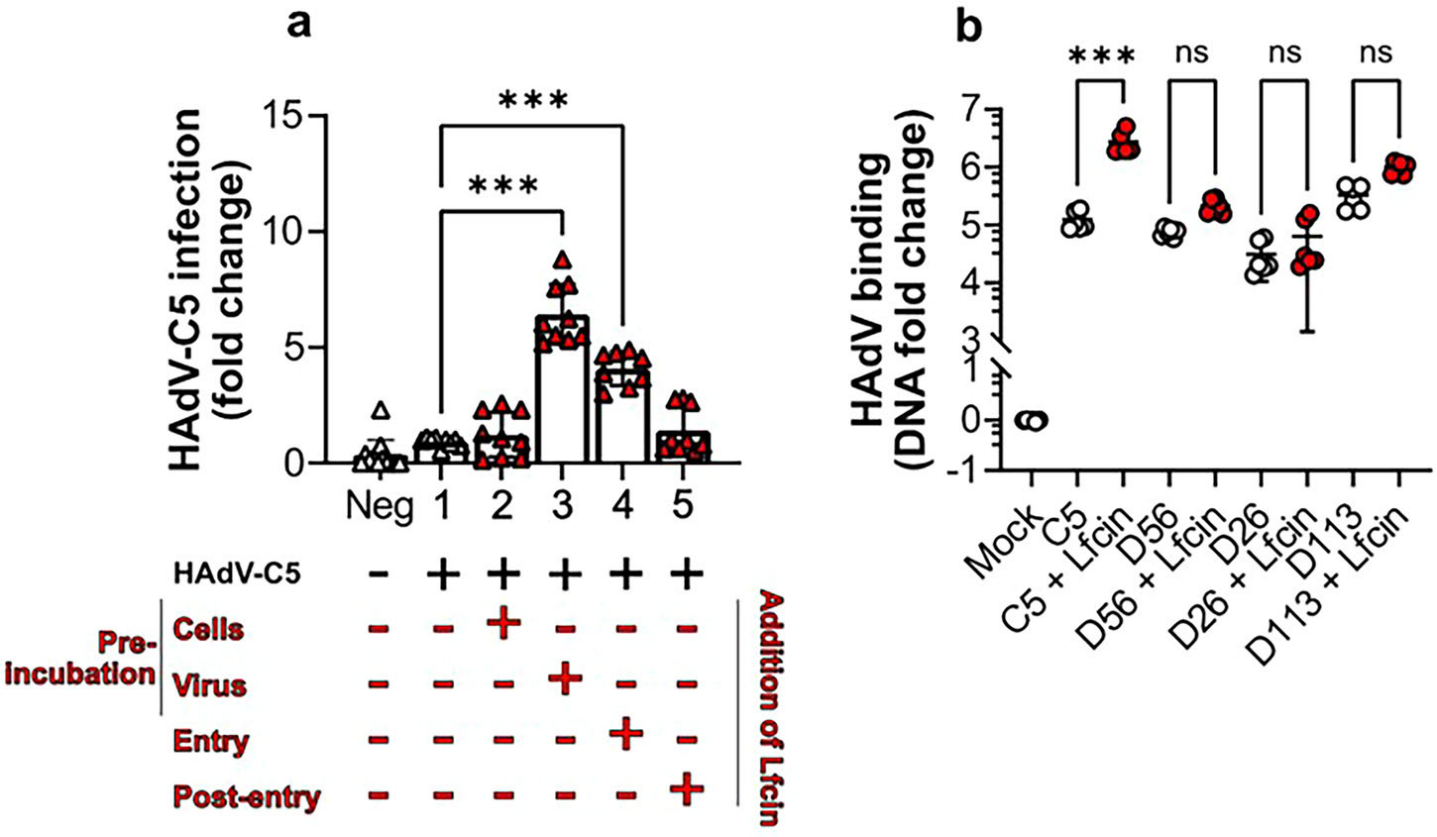
Lfcin promotes HAdV-C5 attachment to and entry into human skeletal myoblasts. **a** Analysis of HAdV-C5 infection (6000 vp/cell) of myoblasts, Lfcin added at various times during the inoculation procedure; 1), HAdV-C5 infection only; 2), Lfcin added to cells for 30 min, then removed (without washing) before HAdV-C5 inoculation; 3), HAdV-C5 pre-incubated with Lfcin 30 min prior to inoculation on cells; 4), HAdV-C5 and Lfcin inoculated on cells simultaneously; or 5), Lfcin added to medium for 48 h after HAdV-C5 inoculation. Samples were stained for HAdV capsid protein and cell nuclei. **b** qPCR-based analysis of HAdV binding to myoblasts with or without Lfcin. 5000 vp/cell were incubated with 2 µM Lfcin and added to cells on ice for 30 min. Binding is normalised against GAPDH and is presented as fold change of viral DNA compared to mock-treated cells. Data are from three independent experiments, presented as mean ± SD. Statistical significance was determined with one-way ANOVA; ns, not significant; *** *P* < 0.001.

As Lfcin interacts with the hexon of HAdV-C5^34^, we anticipated that this interaction is important during virus entry into cells. To test this, we incubated Lfcin with cells and/or virions at different conditions. Pre-incubating Lfcin with myoblasts prior to HAdV-C5 inoculation did not affect the infection (Fig. 3a). However, we observed a significant increased infection when Lfcin and HAdV-C5 were co-incubated with myoblasts, and infection was maximally enhanced when Lfcin was preincubated with HAdV-C5 before incubation with myoblasts (Fig. 3a, Supplementary Fig. 4). The relative percent increase in infection when HAdV-C5 was pre-incubated with Lfcin was around 50%. Adding Lfcin to myoblasts after inoculation did not alter infection. To further investigate if Lfcin facilities infection at an early stage, we quantified HAdV-C5 binding to myoblasts by qPCR. In the presence of Lfcin, we observed a 20-fold increase in cell-bound HAdV-C5 genomes, as compared to binding without Lfcin. In contrast, Lfcin did not affect the binding of HAdV-D26, −D56, or −D113 to myoblasts (Fig. 3b). These results demonstrate that Lfcin enables HAdV-C5 infection by promoting virion attachment to myoblasts.

### Lfcin and FX enable HAdV-C5 infection of human skeletal myoblasts through different mechanisms

**Fig. 4:**
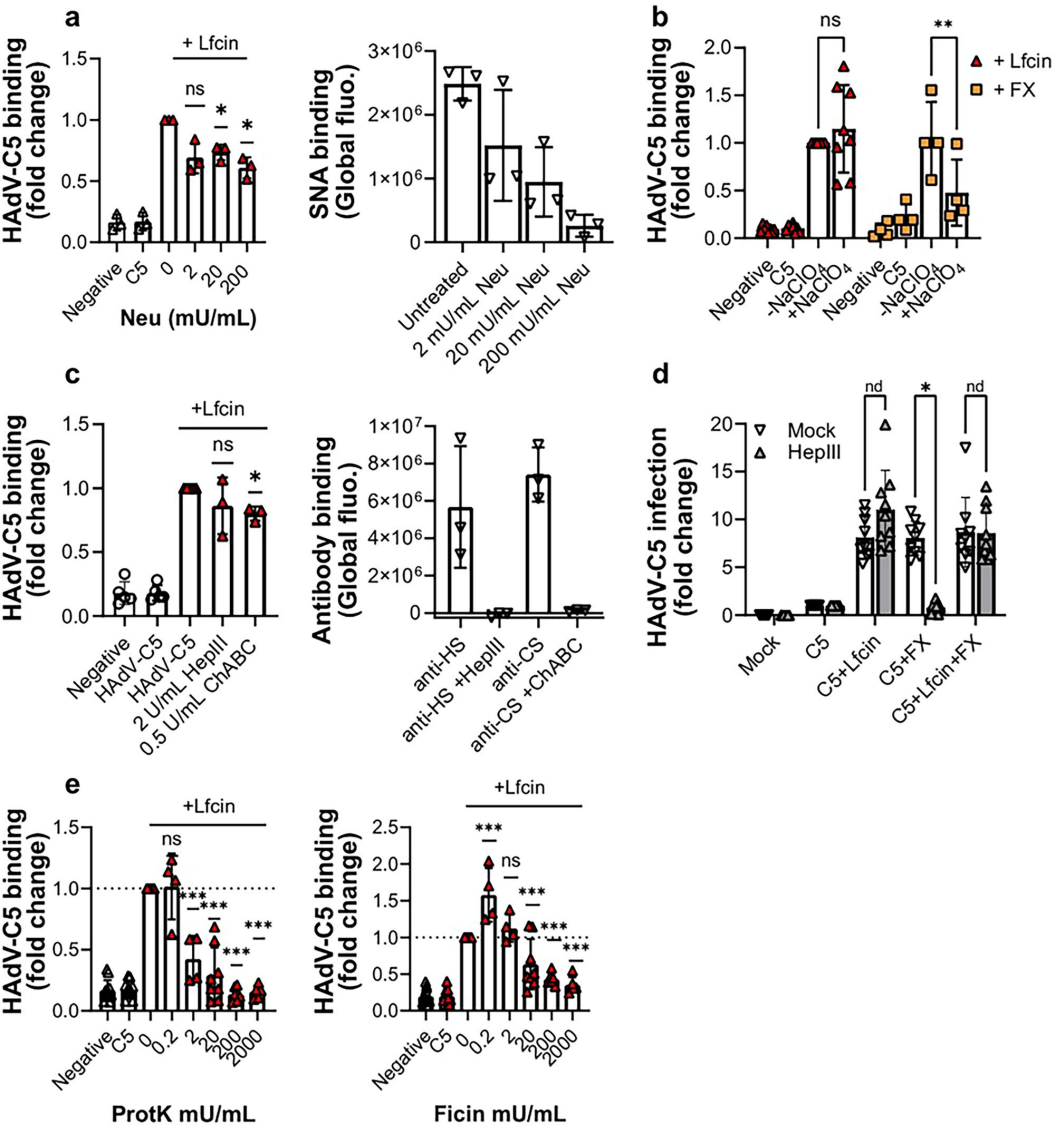
Lfcin enables binding to myoblasts is reduced dose-dependently by protease treatment of, but not by glycan removal. Flow cytometry-based virus-cell binding assay of Alexa Fluor 488-labelled HAdV-C5 binding to myoblasts cells treated with (**a**). neuraminidase from Vibrio Cholerae, or (**b**). sodium perchlorate, **c** glycosidases heparinase III or chondroitinase ABC. **d** Analysis of HAdV-C5 infection in the presence of 2 µM Lfcin and/or 10 µg/mL Factor X (FX) on myoblasts cells, stained for HAdV capsid protein and cell nuclei. **e** Flow cytometry-based analysis of Alexa Fluor 488-labelled HAdV-C5 binding to myoblasts cells treated with proteases Ficin or Proteinase K at 0.2 mU – 2000 mU/mL. Verification of sialic acid removal was done with lectin staining, and for glycosaminoglycans with antibody staining. Binding is presented as fold change of Lfcin control. Data are from at least two independent experiments, presented as mean ± SD. In **a**, **b**, and **e** statistical significance was determined with unpaired T-test; ns, not significant; *P < 0.03; ***P < 0.001. In **c**, and **d** statistical significance was determined with one-way ANOVA; nd, not determined; * *P* < 0.03.

Systemic delivery of HAdV-C5-based vectors results in FX engaging with the hexon, which targets the virus to heparan sulfate-rich regions, mainly on hepatocytes^19,51^. Whereas Lfcin is likely to bind to HVR-1, FX primarily interacts with HVR5 and 7^52^ implying that these molecules mediate HAdV-C5 entry through different mechanisms. We initially sought to understand the nature of the cell surface molecules engaged in Lfcin-mediated binding to myoblasts by investigating the role of negatively charged glycans. Neuraminidase treatment, which removes sialic acid-containing monosaccharides from the cell surface, did not affect Lfcin-mediated binding of HAdV-C5, but reduced binding of the sialic acid-binding lectin (SNA) control (Fig. 4a, Supplementary Fig. 5a). Similarly, neither did the removal of sulfate groups by NaClO4 treatment (Fig. 4b, Supplementary Fig. 5b) nor enzymatic removal of glycosaminoglycans (GAGs) heparan sulfate or chondroitin sulfate significantly affect Lfcin-mediated binding, although the respective controls were affected as expected (Fig. 4c, Supplementary Fig. 5c). In contrast, removal of sulfate groups and heparan sulfate significantly reduced FX-mediated HAdV-C5 binding, suggesting that Lfcin enables HAdV-C5 binding to myoblasts through a mechanism distinct from FX. To confirm this, we preincubated HAdV-C5 with Lfcin, FX, or both, and quantified infection in heparinase III-treated myoblasts. Heparinase III treatment reduced FX-mediated infection but had no effect on Lfcin-mediated infection. In the presence of both factors, infection levels remained comparable to those seen with Lfcin alone, further supporting that Lfcin-mediated infection occurs independently of heparan sulfate (Fig. 4d, Supplementary Fig. 5d). Treatment with the proteases Ficin or Proteinase K resulted in a significant, dose-dependent decrease in Lfcin-mediated HAdV-C5 binding (Fig. 4e, Supplementary Fig. 5e), suggesting that one or more protein component(s) are involved in Lfcin-mediated HAdV-C5 binding to these cells. Taken together, these results suggest that Lfcin and FX enable HAdV-C5 infection of myoblasts through distinct mechanisms, and that the respective complex binds to different cell surface molecules.

### Lfcin is a more potent enabler of HAdV-C5 infection and less toxic than other positively charged molecules

**Fig. 5:**
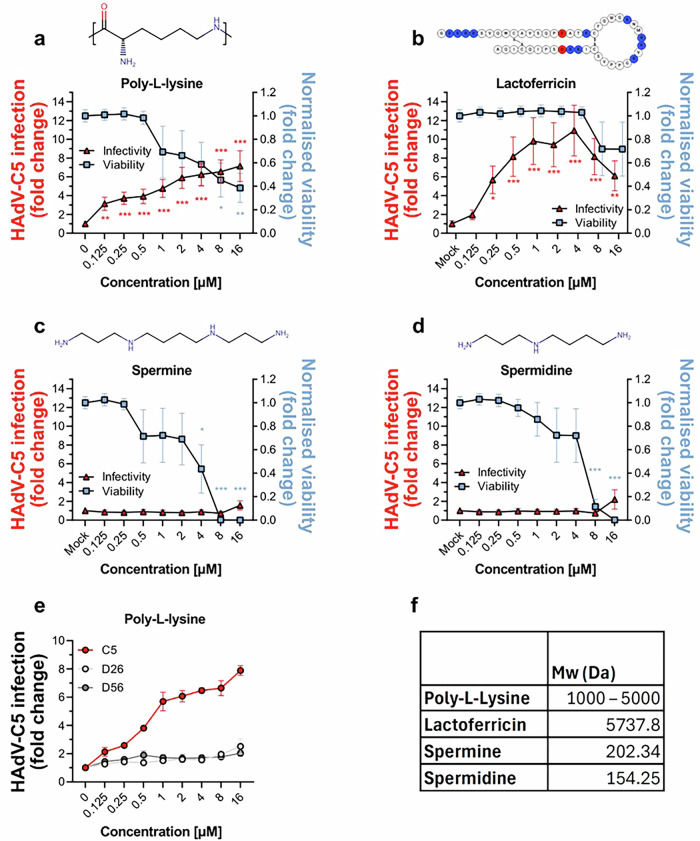
Lfcin shows less cytotoxicity in myoblasts compared to other positively charged molecules. **a**–**d** Molecular structures of poly-l-lysine, spermine, spermidine, and amino acid sequence of Lfcin. Analysis of HAdV-C5 infection (6000vp/cell) and cell viability in the presence of increasing concentrations of (**a**). poly-L-lysine, **b**. Lfcin, **c**. spermine, and (**d**). spermidine. **e** Analysis of infection with HAdV-C5 (6000 vp/cell), −D26 (12,000 vp/cell), and −D56 (40,000 vp/cell) in the presence of increasing concentrations of PLL, ranging from 0.125 to 16 µM. **f** Molecular weights (Da) of (Lfcin), poly-L-lysine, spermine, and spermidine. In infection experiments, myoblasts were stained for HAdV capsid protein and cell nuclei. Viability was measured with Celltiter Glo ATP assay (Promega). Data are from three independent experiments, presented as mean ± SD. Statistical significance was determined with unpaired T-test; *, *P* < 0.03; **, *P* < 0.002; *** *P*, < 0.001.

Dose-dependent experiments demonstrated increased HAdV-C5 infection of myoblasts starting at 0.125 µM Lfcin with a peak around 1-4 µM, followed by decreased infection at 8 and 16 µM (Fig. 5b). Since other positively charged molecules, such as poly-L-lysine (PLL), spermine, and spermidine, can also enhance HAdV-C5 infection of various cell types^24,28^, we compared their ability to enable infection in myoblasts with that of Lfcin. Pre-incubation of HAdV-C5 with PLL (Fig. 5a) increased infection but less efficient than Lfcin (Fig. 5b). In contrast, neither spermine nor spermidine altered infectivity (Fig. 5c, d). Given that positively charged polymers such as PLL are known to be cytotoxic at high concentrations^53^, we assessed the cytotoxicity of each compound using an ATP-based assay as proxy for cell viability. PLL was toxic at concentrations above 0.5 µM (Fig. 5a) whereas Lfcin remained non-toxic at concentrations up to 8 µM (Fig. 5b). Spermine and spermidine both showed signs of cytotoxicity at concentrations above 0.25 µM and 2 µM, respectively (Fig. 5c, d). Since PLL increased HAdV-C5 infection, we tested whether this effect could be applied to other HAdV types. Only a minor increase in infection was observed for HAdV-D26 and −D56 (approximately 2-fold; Fig. 5e), compared to the 8-fold increase seen with HAdV-C5 at peak concentration. These results further support that certain – but not all – positively charged molecules enable infection of HAdV-C5 in myoblasts.

### Lfcin-mediated aggregation of HAdV-C5 particles at high concentrations correlates with reduced infection

**Fig. 6:**
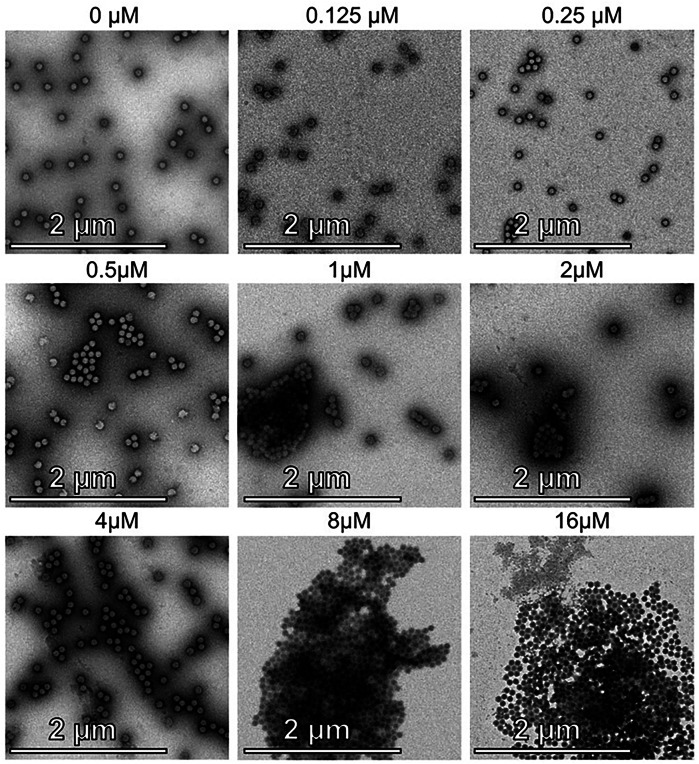
Negative stain EM of HAdV-C5 in the presence of increasing concentrations of Lfcin. Negative stain EM analysis of HAdV-C5 virus particles with an increasing concentration of Lfcin. All samples were prepared with the same amount of virus particles, with Lfcin concentrations ranging from 0.125 µM to 16 µM in 2-fold increments, as indicated above each image.

To determine the underlying cause of the decreased infection observed at Lfcin concentrations ≥8 µM (Fig. 5b), we performed negative stain EM imaging of HAdV-C5 in the presence of increasing Lfcin concentrations (0.125 µM–16 µM in 2-fold increments). In the untreated sample, virus particles appeared almost exclusively as single particles (Fig. 6). At low Lfcin concentrations up to 4 µM no major changes in virus particle distribution were observed, but smaller clusters <1 µm in size formed along with single virus particles. At 4 µM, some larger clusters (~4 µm in diameter) formed, although single virus particles were still abundant. However, at higher concentrations (8 µM–16 µM) extensive clustering was observed with substantially fewer single particles visible (Fig. 6). The reduction of free virions at high Lfcin concentrations correlated with the decreased infection efficiency seen in Fig. 5b.

### Lfcin reduces serum-mediated neutralization of HAdV-C5 infection

**Fig. 7:**
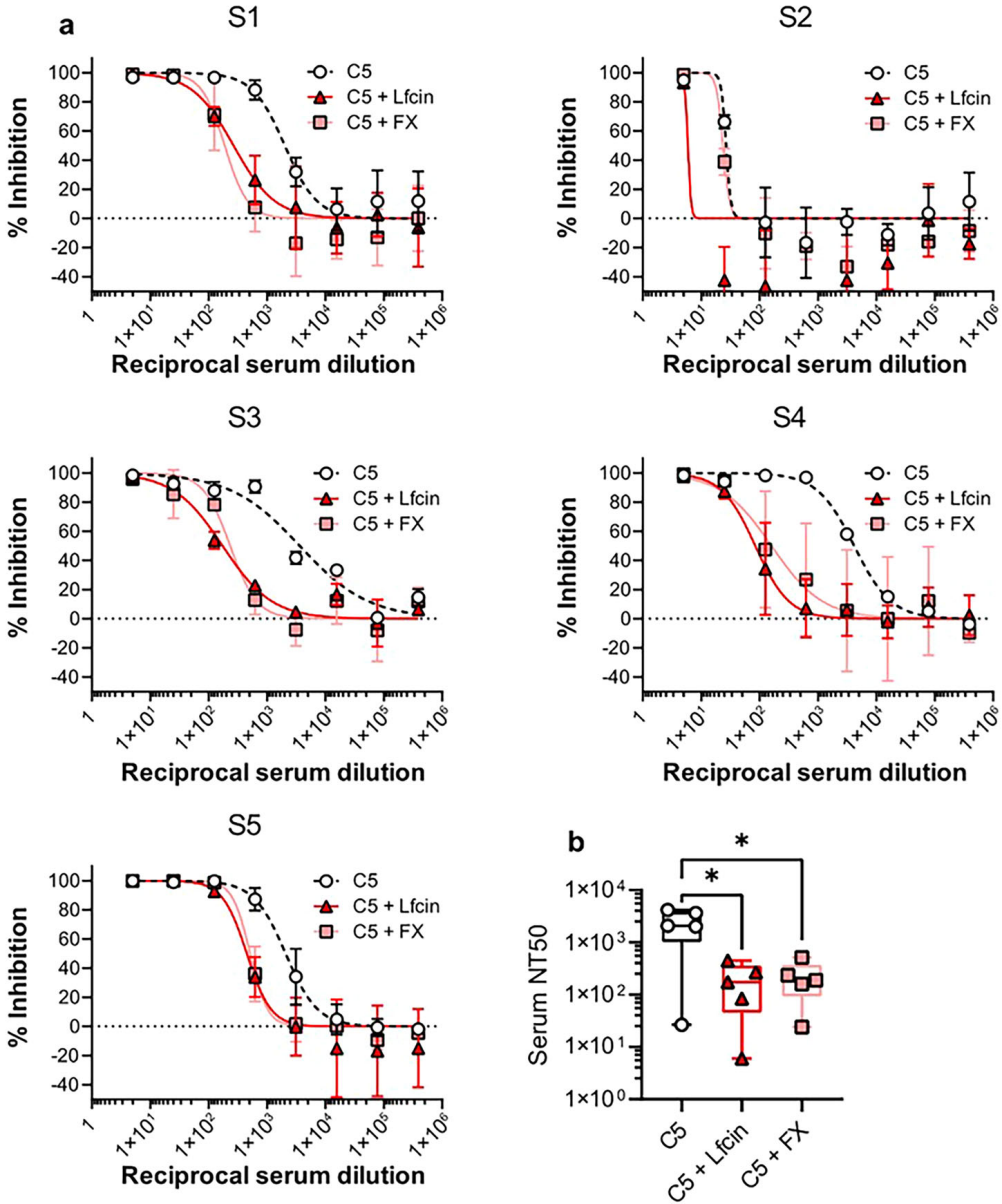
Reduced serum neutralization of HAdV-C5 in the presence of Lfcin. **a** Analysis of HAdV-C5 neutralization on A549 cells. HAdV-C5 (50 000 vp/cell) was preincubated with Lfcin (2 μM) or FX (10 μg/mL) and subsequently incubated with heat-inactivated HAdV-C5 neutralizing serum before infection of A549 cells. Data are from two independent experiments, presented as mean ± SD. **b** Average NT50 of individual sera on HAdV-C5 infection in the presence of Lfcin or FX. Statistical significance was determined by the Wilcoxon test for nonparametric distribution (∗, *P* < 0.03). Medians are shown as lines within each bar, calculated with GraphPad 10.4.0.

Since FX-binding to hexon can protect virions from neutralization by pre-existing antibodies, we investigated if Lfcin exerted a similar activity. HAdV-C5 seroprevalence is estimated to be about 50% in Europe (reviewed by Mennechet et al. 2019^9^), therefore, we selected sera from a Swedish COVID-19 vaccination cohort (CoVacc; see Material and Methods section for details) to evaluate this. Given the low baseline HAdV-C5 infection of myoblasts cells in the absence of Lfcin, we performed neutralization assays in A549 cells. Sera with neutralizing properties against HAdV-C5 were used in infection experiments in the presence of Lfcin or FX. We analysed the capacity of serums to neutralize HAdV-C5 and observed a general reduction in neutralization potency of sera when Lfcin was present, comparable to the effect of FX (Fig. 7a). The neutralization capacity was calculated for each serum as 50% neutralizing titre (NT50). On average, Lfcin reduced serum NT50 close to 20-fold, which was comparable to FX (Fig. 7b).

## Discussion

In this study, we investigated the role of Lfcin during HAdV-C5 infection of human skeletal muscle cells. We demonstrate that Lfcin enables HAdV-C5 cell entry and infection through a mechanism that is independent from the canonical receptor CAR (Figs. 1–3), suggesting a potential strategy for improving adenoviral vector-based gene delivery and immunization approaches.

Lfcin, along with its full-length protein lactoferrin, enhances species C HAdV infection of epithelial cells, T cells, and dendritic cells^33–35^, but the effect of Lfcin has not previously been evaluated for infection/transduction of muscle cells by AdVs. Our results demonstrates that Lfcin significantly enables HAdV-C5 infection/transduction of both proliferating myoblasts (Fig. 1) and terminally differentiated myotubes (Fig. 2), which suggests that inclusion of Lfcin can be useful for clinical applications of HAdV-C5-based vectors targeting. Such vectors naturally display low transduction efficiency in CAR-deficient cell lines, and ageing myotubes are even more resilient to AdV transduction^29^. Therefore, strategies that improve transduction are of particular interest for gene therapy and immunization applications targeting muscle tissue. By identifying Lfcin as a naturally occurring peptide that enables HAdV-C type infection, and characterization of the underlying molecular mechanism, our study adds to prior efforts aimed at improving vector transduction in muscle cells^24,26,27,54^. Additionally, due to its non-toxic (endogenous) nature in humans, lactoferrin has been used in clinical trials for the treatment of conditions including anaemia^55,56^, gastrointestinal and respiratory symptoms in infants^57^, and mortality in preterm infants^58,59^. Given its established safety profile, Lfcin could represent a promising candidate for improving viral vector administration with reduced risk compared to synthetic polymers^53,60,61^.

We have previously shown that replacing HVR1 of HAdV-C5 with that of HAdV-A31 abolishes the infection-enhabling effect of Lfcin in CAR-negative cells^34^. HVR1 is remarkably longer and more negatively charged in species C compared to other HAdV species (Fig. 1e) and is implicated in the interaction with the positively charged Lfcin. Notably, even within species C, we observed differences in Lfcin-mediated infection; Lfcin enabled infection of HAdV-C1 and −C2 more efficient than HAdV-C5 and −C6 (Fig. 1c), which correlates with HAdV-C1 and −C2 having more negatively charged residues in their HVR1. This is also in agreement with our finding that Lfcin act during the binding step and requires direct interaction with the virion for maximal efficiency (Fig. 3). Engaging Lfcin for more efficient targeting of muscle cells in vaccination and gene therapy is likely to be specific for species C-based vectors and will not enhance the efficacy of vectors based on e.g., HAdV-D26 and ChAdOx. Another proposed charge-dependent interaction of potential relevance for vaccination is the interaction between ChAdOx hexon protein and platelet factor 4, which is associated with adverse effects. Although Lfcin-mediated binding is abolished upon protease treatment of myoblasts (Fig. 4e), we still do not understand mechanistically how Lfcin enable CAR-independent binding of HAdV-C5 to muscle cells. Lfcin contains several charged residues (Arg and Lys), which are potentially capable of mediating charge-dependent binding. Our data show that individual glycan removal did not significantly alter Lfcin-mediated HAdV-C5 binding (Fig. 4a–c), but such treatment reduced FX-mediated binding (Fig. 4d). This suggests that Lfcin and FX engage distinct cellular receptors for entry and infection. Interestingly, preincubating HAdV-C5 with Lfcin followed by FX did not enable myoblast infection beyond the effect of either factor alone, suggesting that Lfcin may interfere with FX binding to the viral capsid. Given that FX mediates liver sequestration of HAdV-C5-based vectors, when delivered intravenously^62^, further investigations are needed to determine if and how Lfcin affects distribution upon systemic delivery.

We assessed whether other charged molecules including PLL, spermine, and spermidine could replicate the effect of Lfcin in enhancing HAdV-C5 infection of muscle cells. While spermine and spermidine enhance HAdV-C5 vector transduction in mesenchymal stem cells^28^, we observed no such effect in muscle cells (Fig. 5). Instead, only PLL enabled infection of HAdV-C5. Notably, the molecular weights of both spermine (202.34 Da) and spermidine (154.25 Da) are considerably lower than those to Lfcin (5737.8 Da) and PLL (a mixture of polymers ranging from 1000 to 5000 Da) (Fig. 5f). This suggests that molecular size may play a mechanistic role facilitating interaction between virus and muscle cells, or that the relevant factors necessary for infectious uptake are not present on muscle cells. Polymers have been evaluated as viral transduction enhancers, however their heterogenous size and higher cytotoxicity demonstrates unpredictability^53,61^. Interestingly, we observed a reduction in Lfcin-mediated infection at higher concentrations (≥4 µM), which correlated with virus particle clustering at high concentrations of Lfcin. Given that full-length lactoferrin binds to and interacts with several host factors and infectious agents, Lfcin may share this property, leading to aggregation of viral particles at elevated concentrations. In negative stain EM, we observe clustering starting at 0,5 µM Lfcin (Fig. 6), which becomes more prominent up to 8- and 16 µM Lfcin, where most if not all particles are clustered. These findings suggest that while Lfcin enables HAdV-C5 transduction of cells, the effect is concentration-dependent. If used as a vector enhancer, careful concentration optimization would be essential to maximize its transduction-enhancing properties.

Studies estimate that over half of adults worldwide have neutralizing antibodies against HAdV-C5^9,63,64^, posing a significant challenge to HAdV-C5-based gene therapy. The anti-hexon antibodies are directed against the HVRs, which together form the “tower” regions on the surface of the hexon^12,65,66^. Previous attempts to avoid neutralizing antibodies have been done by shielding the hexon epitopes with scFv fragments^67^. It has also been shown that FX bound to the HAdV-C5 capsid prevents neutralizing antibodies^20^. Our study suggests that natural peptides such as Lfcin might provide a simple solution for mitigating inhibitory effects of neutralizing antibodies (Fig. 7) while enhancing infection in CAR-deficient cells. Building on findings that muscle-targeting peptides improve viral targeting via hexon insertion^27^, direct Lfcin incorporation into the capsid may also offer enhanced vector efficiency and expand tropism to other CAR-negative cells, such as leucocytes.

Apart from being present in many body fluids, lactoferrin is secreted by neutrophils, which are present in e.g., tonsils and adenoids and are recruited to sites of inflammation^36,39^. This correlates with the unique ability of species C HAdVs to persistently infect T lymphocytes within the tonsils and adenoids^68,69^, despite their lack of CAR expression. As lactoferrin can enhance uptake in several types of cells^33–35^ it is tempting to hypothesise that species C HAdV have evolved a unique – among both human and non-human AdV – ability to utilize lactoferrin and/or Lfcin for T-cell uptake in vivo. We suggest and demonstrate that this capacity can be utilized for efficient transduction of CAR-deficient cells such as muscle cells, by HAdV-C types.

In summary, our study identifies Lfcin as a molecule that efficiently enables HAdV-C5 infection in muscle cells, acting through a CAR-independent mechanism. By promoting viral binding to and entry into cells and simultaneously reduce neutralization by pre-existing antibodies, Lfcin presents a promising tool for improving adenoviral vector efficacy. Future studies should focus on elucidating the specific host receptor(s) involved in Lfcin-mediated uptake, as well as study the effects of Lfcin in systemic delivery. Additionally, exploring Lfcin insertion into the viral capsid could provide a means to enhance targeting specificity while mitigating pre-existing immunity. Together, these findings pave the way for novel strategies to optimize adenoviral vector applications in gene therapy and vaccination.

## Methods

### Cell lines, viruses, antibodies and compounds

A549 cells (human lung epithelial carcinoma: ATCC CCL-185) were maintained in DPH (Dulbecco’s modified eagle medium (Sigma-Aldrich) with 100 µg/mL Penicillin + 100 U/mL Streptomycin (Gibco) and 20 mM Hepes (Fisher)) supplemented with 10% foetal bovine serum (FBS) (HyClone). The human skeletal myoblast cell line MDC1A;B38/03-CT4^70^ was cultured in skeletal muscle cell growth medium (Provitro, 201 0602). For differentiation 20,000 cells/well were seeded into black 96-well flat-bottom plates with transparent bottom. After 24 h the medium was changed to differentiation medium (DPH supplemented with 2% horse serum (Sigma-Aldrich)), after which the cells were allowed to differentiate for 5 days, with exchange to fresh differentiation medium every day.

Wildtype human adenoviruses, HAdV-C5 (strain Adenoid 75), and HAdV-D26 (strain BP-2), D56 (wildtype, purchased from ATCC), and D113 (strain 212) were propagated in A549 cells and purified on a Caesium chloride (CsCl) gradient as described previously^71^ except for purified and desalted virus being harvested in PBS supplemented with 10% glycerol. Purified virions were stored at −80 °C until further use. HAdV-C5 was labelled with an Alexa Fluor 488 NHS ester (A20000, Thermo Fisher) according to manufacturer’s instructions. HAdV-C5-eGFP was obtained from Vector Development Laboratory. HAdV-D56-eGFP and HAdV-D113-eGFP were obtained from Batavia Biosciences, ChAdOx1-SARS-CoV2-Spike was a kind gift from Professor Alan Parker at Cardiff University.

A pan-adenovirus antibody detecting the capsid protein was purchased from Merck Millipore (MAB8052 clone 20/11). Alexa Fluor 647-conjugated secondary antibodies from Invitrogen. Poly-L-Lysine (P0879, 1000-5000 kDa), spermine (S4264), and spermidine (S2626) were all purchased from Sigma-Aldrich. Human Lfcin 1-49 was obtained from the Center for Food Technology, processed by Hamilton, Qld, Australia. In brief, human milk from multiple donors was collected and lactoferrin was purified. Lfcin was generated via pepsin cleavage and further purified as described previously^72^.

Human sera for neutralization assays were obtained via CoVacc, an open multicentre phase IV study of the immune response to vaccination against COVID-19 (Ethical approval by the Swedish Ethics Review Authority, Dnr 2021-00055, and registered at European Clinical Trials Database EUDRACT Number 2021-000683-30)^73^.

### Infection experiments with wild-type HAdVs

A549 and myoblasts were seeded at 20,000 cells/well into black 96-well flat bottom plates with transparent bottom. After 24 h, wild-type HAdV were incubated with 2 µM Lfcin or 10 µg/mL FX on ice for 30 min, added to cells and incubated at 37 °C. The virus amount was titrated on MDC1A cells to give 10% baseline infection. After 60 min, unbound virus-Lfcin complex was washed away using DPH, and DPH supplemented with 10% FBS was added.

Infection with wt HAdV was stopped after 44 h. The cells were fixed using 4% paraformaldehyde (PFA) for 5 min at room temperature, and permeabilised with 100% MeOH for 10 min at −20 °C. Cells were stained with an anti-HAdV capsid protein monoclonal antibody MAB8052 (Millipore). An Alexa Fluor 647-conjugated secondary antibody was used for detection. To ensure an intact monolayer, the cells were stained with Hoechst 3342 diluted 1:10,000. The cells were fixed using 4% PFA and stained with Hoechst 1:10 000. In all experiments, analysis was performed on a Cytation5 (BioTek) instrument. Imaging and analysis were performed using a Cytation 5 instrument (BioTek) at 4x magnification. Fluorescence signals from Hoechst (nuclei) and Alexa Fluor 647 (infected cells) were acquired, with exposure settings adjusted based on positive controls. The total number of cells was determined by nuclear staining, and infected cells were identified based on the capsid protein signal. Percent infection was calculated as the number of capsid-positive cells divided by the total number of nuclei in each well. Fold change was calculated based on percent infection, by dividing the value obtained in the presence of Lfcin by the value obtained in its absence.

### Transduction experiments with HAdV vectors

A549 and myoblasts were seeded at 20,000 cells/well into black 96-well flat-bottom plates with transparent bottom. After 24 h, AdV vectors (expressing GFP or SARS-CoV-2 spike protein) were incubated with 2 µM Lfcin or 10 µg/mL FX on ice for 30 min, added to cells and incubated at 37 °C. Virus amount was titrated on MDC1A cells to give 10% baseline transduction. After 60 min, the unbound virus-Lfcin complex was washed away using DPH, and DPH supplemented with 10% FBS was added.

Transduction with AdV vectors was stopped after 24 h, and the cells were fixed using 4% paraformaldehyde (PFA) for 5 min at room temperature. To ensure an intact monolayer, the cells were stained with Hoechst 3342 diluted 1:10,000. In all experiments, analysis was performed on a Cytation5 (BioTek) instrument as described above.

### Virus-cell binding experiments

A549 or myoblasts were seeded at a density of 200,000 cells/well in 12-well cell culture plates (VWR, Avantor). After 24 hours, confluent monolayers were rinsed with cold DPH. On ice, HAdVs were incubated with 2 µM Lfcin. After 30 min incubation, 5000 vp/cell of HAdV-Lfcin in DPH was added to cells. After 30 min incubation, cells were rinsed three times with cold PBS. Samples were harvested by cell lysis and total DNA was isolated using a NucleoSpin Tissue DNA extraction kit (Macherey-Nagel, 740952). Bound HAdV genomes were quantified by qPCR using qPCRBIO Probe Mix Lo-Rox (QPCRBIO, PB20.21). PCR conditions being 1 cycle of 95 °C for 2 minutes, 40 cycles of 95 °C for 5 seconds and 65 °C for 30 seconds. Relative gene expression was calculated by 2-ΔΔCT where the HAdV gene expression was normalised against GAPDH expression. Primers and probe detecting vial hexon were: 5’-CWTACATGCACATCKCSGG-3’ forward primer; 5’-CRCGGGCRAAYTGCACCAG-3’ reverse primer; 5’-[6FAM]-AGGACGCCTCGGAGTACCTGAGCCCCG-[TAMRA]-3’ probe, and for detection of GAPDH the TaqMan Gene Expression Assay kit was used (Thermo Fisher, 4331182, Hs02758991_g1).

### Flow cytometry experiments

Myoblasts were detached using PBS with 0.05% EDTA and transferred to a falcon tube. Cells were pelleted at 1500 rpm for 3 min and the pellet was resuspended in growth medium and allowed to reactivate for 60 min at 37 °C on a tipping board. Cells were treated with the following variations of enzymes or compounds in binding experiments:

1) Neuraminidase from Vibrio Cholerae (Sigma, 72197) at 2, 20, or 200 mU/mL, Heparinase III (Merck, H8891) at 2 U/mL, or Chondroitinase ABC (Sigma, C3667) at 0.5 U/mL for 1 h in DPH at 37 °C with agitation.

2) Sodium perchlorate (NaClO4) at 50 µM for 24 h in growth medium at 37 °C.

3) Ficin (Sigma, F4125) or Proteinase K (Sigma, 1073930010) in 10-fold dilution from 0.2–2000 mU/mL for 30 min in DPH at 37 °C with agitation.

Subsequent steps were performed on ice. Alexa Fluor 488-labelled HAdV-C5 was incubated with 2 µM Lfcin. After 30 min cells were transferred to a 96 well v-bottom plate at 200,000 cells/well and washed with DPH before virus-Lfcin complex was added. After 60 min incubation with agitation, cells were washed once and resuspended in PF buffer (PBS with 2% FBS)

Single wells were stained for cell surface expression of DSG-2, CAR, CD46 or sialic acid using respectively either mouse IgG anti-DSG2 (Santa Cruz Biotechnology AH12.2) 1:100, mouse IgG anti-CAR (Merck 05-644) 1:2000, mouse IgG2ak anti-CD46(MCP)-FITC (Ancell 197-040) 1:50, Sambucus Nigra lectin (SNA) conjugated with biotin (Vector laboratories B1305) 1:500, mouse IgM anti-chondroitin sulfate (C8035, Sigma-Aldrich) 1:500, or mouse IgM anti-heparan sulfate (370255-S, AMSbio) 1:1000 diluted in PF buffer for 30 minutes on ice. After washing away unbound antibodies with PF buffer cells were incubated with either an Alexa Fluor-488 conjugated anti-mouse IgG/IgM (H + L) secondary antibody (Invitrogen) diluted 1:1000, or Alexa Fluor-488 conjugated streptavidin (Invitrogen) diluted 1:1000 in PF buffer for 30 minutes on ice.

All samples were analysed for fluorescence intensity using a ZE5 flow cytometer (BioRad) instrument. Gating was done for single cells based on side scatter and in each sample 10,000 events were recorded and analysed for geometric mean fluorescence intensity.

### Effect of positively charged molecules on cell viability

Myoblasts cells were seeded at 20,000 cells/well in white opaque 96 well flat bottom plates. After 24 h, Lfcin, PLL, spermine, or spermidine was added in 2-fold dilutions ranging from 0.0625–16 µM in 100 µL to individual wells and the plate was incubated for 60 min at 37 °C. Cell viability was measured by ATP production using the CelltiterGlo Luminescent Cell Viability Assay kit (Promega) according to manufacturer’s instructions.

### Negative stain EM

HAdV-C5 was mixed with different concentration of Lfcin (16 µM–0.125 µM in 2-fold dilution) in PBS before addition onto glow-discharged and formvar/carbon coated grids (size 300 mech in copper, Sigma Aldrich). HAdV-C5 without Lfcin was used as a mock. Virus samples were negatively stained with 1,5% uranyl acetate and imaged in with Talos 120 C (FEI, Eindhoven, The Netherlands) operating at 120 kV.

### Neutralization experiments

A549 and myoblasts were seeded at 20,000 cells/well into black 96-well flat-bottom plates with transparent bottom 24 h prior to infection. *Serum from patients immunised against SARS-CoV-2 with one dose of AstraZeneca ChAdOx1 vector followed by a boost with Comirnaty mRNA (7-10 days post dose 2). HAdV-C5 was titrated on MDC1A cells to give 50% baseline infection*. Sera were heat-inactivated by incubation at 56 °C for 10 min. HAdV-C5 was incubated with 2 µM Lfcin or 10 µg/mL FX on ice for 30 min. Sera was titrated in 5-fold dilutions with virus and incubated for 30 min at 37 °C. Cells were washed once with DPH and virus-sera mixtures were added. After 60 min, the unbound virus was washed away using DPH, followed by the addition of DPH supplemented with 10% FBS. Infection was stopped after 44 h and fixation and antibody staining of cells and HAdV was performed as described above. Data was normalised to untreated virus and plotted as log(serum concentration) vs inhibition using [Inhibitor] vs Normalized response function with a variable slope using GraphPad Prism. NT50 was determined as the concentration of serum that gives 50% virus neutralisation.

### Statistics

Experiments were performed at least two times in duplicates or triplicates. Results are expressed as means (SD), and student’s T-test, ANOVA, or Wilcoxon’s test was performed using GraphPad Prism, version 10.3.1 for Windows. *P* values of < 0.03 were considered statistically significant.

## Supplementary information

Supplementary Information

## Data Availability

All relevant data supporting the findings – e.g., minimal datasets necessary to interpret, replicate and build upon the methods and/or findings reported in the article – are available in the paper, and or can be made available along with raw data from the corresponding author upon request.

## Code availability

We used Alphafold (version 3) and APBS (version 3.4.1) to predict HVR1 structures and electrostatic potential. Codes are available from references in supplementary information.
