# Supplementary material for: Lactoferricin enables adenovirus infection of human skeletal muscle cells
Source: Npj Viruses. 2025 Aug 19;3:62. doi: 10.1038/s44298-025-00144-7 (PMC12361444; doi:10.1038/s44298-025-00144-7)
Supplement: Supplementary file 1 — Supplementary Information [file 44298_2025_144_MOESM1_ESM.pdf]

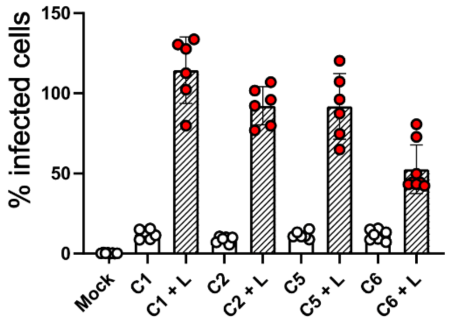


**Supplementary figure 1.** *Analysis of HAdV-C (HAdV-C1 (6000 vp/cell), -C2 (20 000 vp/cell), -C5 (6000 vp/cell), -C6 (20 000 vp/cell)) infection of myoblasts, stained for HAdV capsid protein. Samples were stained for HAdV capsid protein and cell nuclei and data is expressed as percent infected cells, calculated as infected cells divided by total cells. Data are from at least three independent experiments, presented as mean ± SD.*

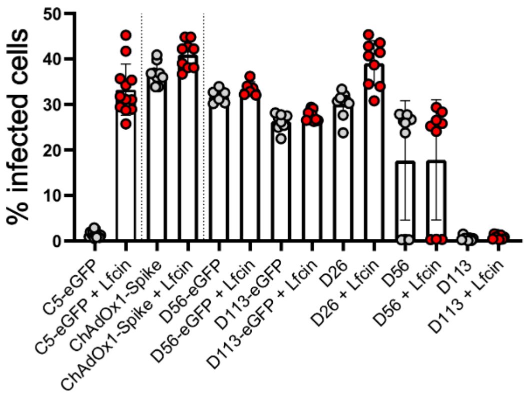


**Supplementary figure 2.** *Analysis of HAdV-D (HAdV-D-26 (12 000 vp/cell), -D56 (40 000 vp/cell), -D113 (30 000 vp/cell)) infection of myoblasts, stained for HAdV capsid protein. Samples were stained for HAdV capsid protein and cell nuclei and data is expressed as percent infected cells, calculated as infected cells divided by total cells. Data are from at least three independent experiments, presented as mean ± SD.*

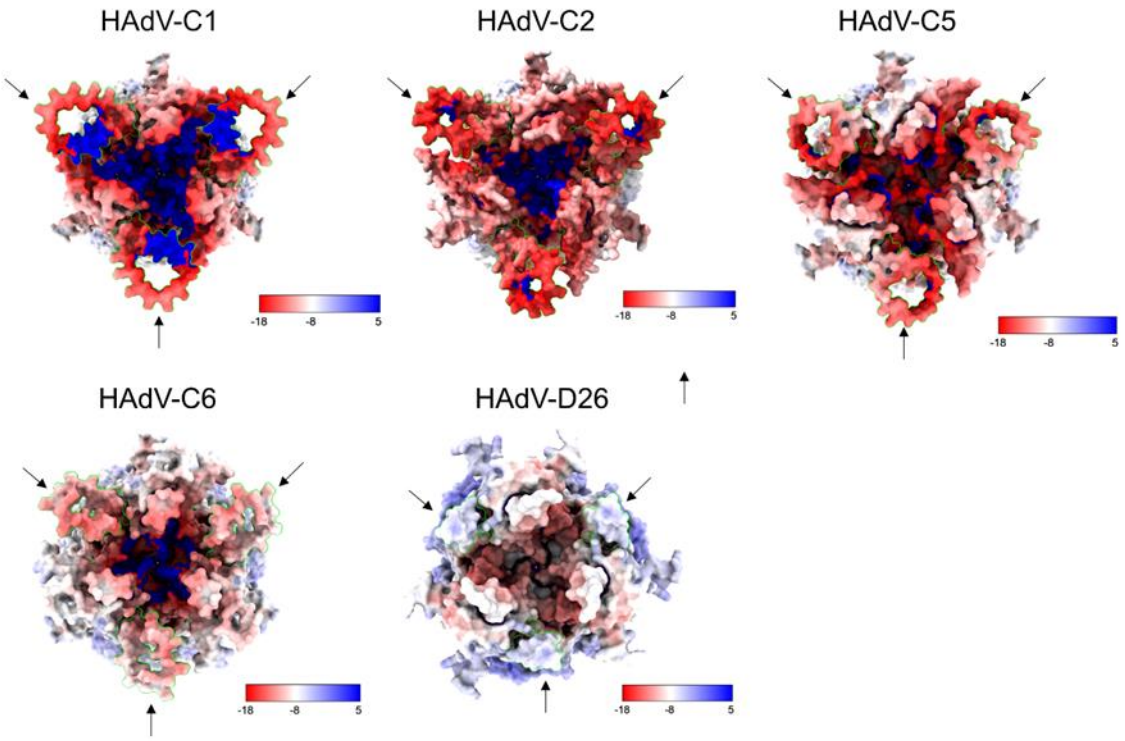


**Supplementary figure 3.** Surface charge prediction of HAdV. The hexon models (the UniProt sequence A0A7D6TSV3 (HAdV-C1), A0A0U5BQA2 (HAdV-C6), PDB files 1p2z (HAdV-C2), 3TG7 (HAdV-C5) and 5TX1 (HAdV-D26)) were used to generate predictions of the HVR1 with AlphaFold3 [1], and the surface electrostatic potential was generated by APBS [2, 3]. Images were rendered using ChimeraX [4]. HVR1 of each hexon model is outlined in green and indicated with arrows, and charge is represented as blue, positive; red, negative; and white, neutral.

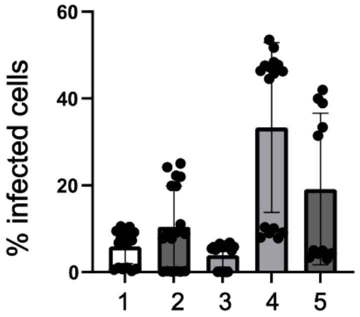


**Supplementary figure 4.** *HAdV-C5 infection (6000 vp/cell) of myoblasts, Lfcin added at various times during the inoculation procedure; 1), HAdV-C5 infection only; 2), Lfcin added to cells for 30 min, then removed (without washing) before HAdV-C5 inoculation; 3), HAdV-C5 pre-incubated with Lfcin 30 min prior to inoculation on cells; 4), HAdV-C5 and Lfcin inoculated on cells simultaneously; or 5), Lfcin added to medium for 48h after HAdV-C5 inoculation. Samples were stained for HAdV capsid protein and cell nuclei and data is expressed as percent infected cells, calculated as infected cells divided by total cells. Data are from three independent experiments, presented as mean ± SD.*

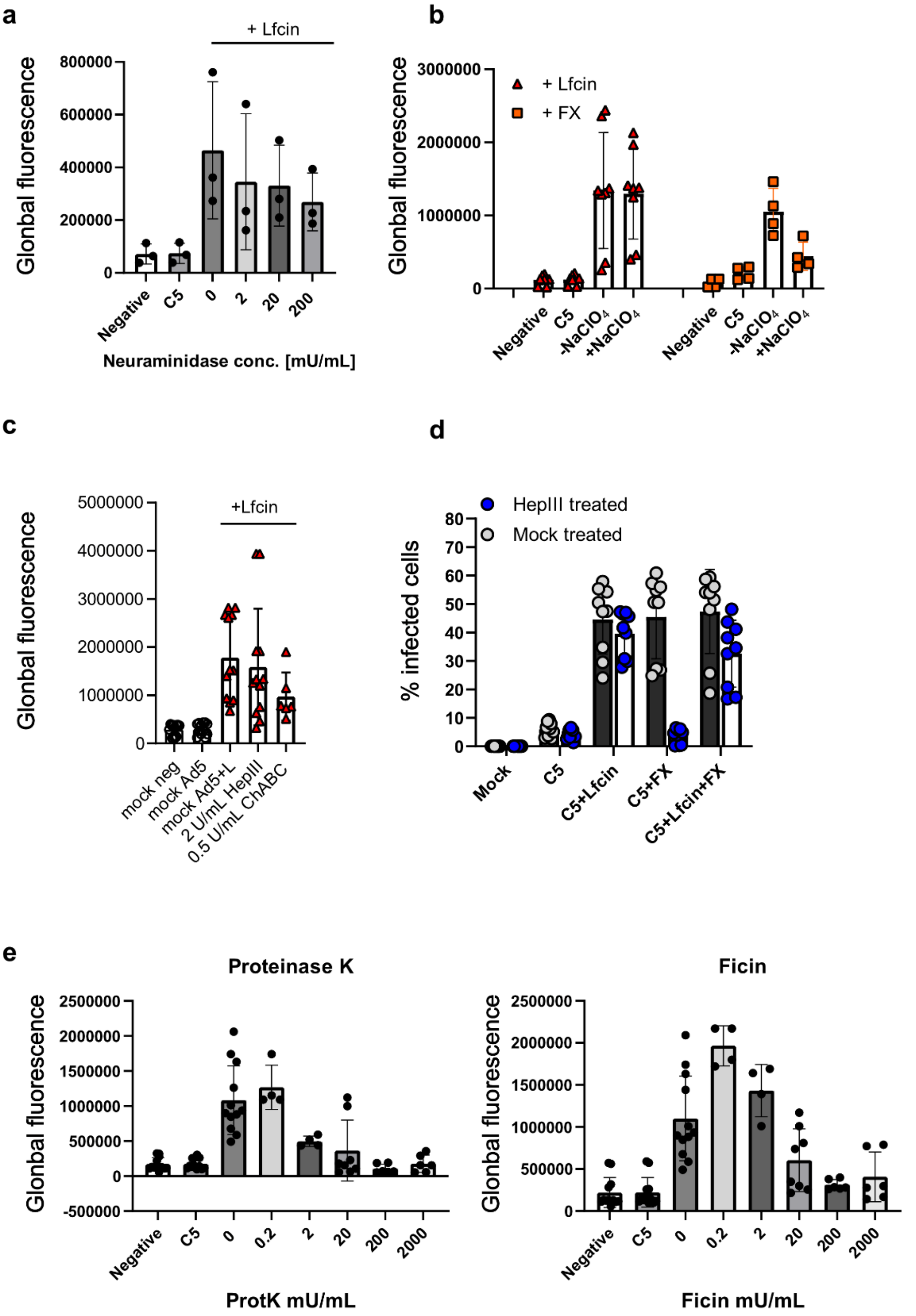


**Supplementary figure 5.** *Flow cytometry-based virus-cell binding assay of Alexa Fluor 488-labelled HAdV-C5 binding to myoblasts cells treated with a. neuraminidase from Vibrio Cholerae, or b. sodium perchlorate, c. glycosidases heparinase III or chondroitinase ABC. d. Analysis of HAdV-C5 infection in the presence of 2 µM Lfcin and/or 10 µg/mL Factor X (FX) on myoblasts cells, stained for HAdV capsid protein and cell nuclei. e. Flow cytometry-based virus-cell binding assay of Alexa Fluor 488-labelled HAdV-C5 binding to myoblasts cells treated with proteases Ficin or Proteinase K at 0.2 mU – 2000 mU/mL. Binding is presented as global fluorescence, infection is presented as percent infected cells, calculated as infected cells divided by total cells. Data are from at least two independent experiments, presented as mean ± SD.*

**Supplementary figure references**

1. Abramson, J., et al., *Accurate structure prediction of biomolecular interactions with AlphaFold 3*. Nature, 2024. **630**(8016): p. 493-500.
2. Jurrus, E., et al., *Improvements to the APBS biomolecular solvation software suite*. Protein Sci, 2018. **27**(1): p. 112-128.
3. Bank, R.E. and M. Holst, *A New Paradigm for Parallel Adaptive Meshing Algorithms*. SIAM Review, 2003. **45**(2): p. 291-323.
4. Pettersen, E.F., et al., *UCSF ChimeraX: Structure visualization for researchers, educators, and developers*. Protein Sci, 2021. **30**(1): p. 70-82.
